# Design, optimization, and in vivo evaluation of invasome-mediated candesartan for the control of diabetes-associated atherosclerosis

**DOI:** 10.1007/s13346-023-01412-w

**Published:** 2023-08-21

**Authors:** Amr Gamal Fouad, Mohammed R. A. Ali, Demiana M. Naguib, Hanan O. Farouk, Mohamed I. Zanaty, Fatma I. Abo El-Ela

**Affiliations:** 1 Department of Pharmaceutics and Industrial Pharmacy, Faculty of Pharmacy, Beni-Suef University, El-Shahid/Shehata Ahmed Hijaz St., Beni-Suef, Egypt; 2 Department of Pharmacology and Toxicology, Faculty of Pharmacy, Beni-Suef University, Beni-Suef, Egypt; 3 Department of Pharmaceutics, Faculty of Pharmacy, Nahda University (NUB), Beni-Suef, Egypt; 4 Department of Pharmaceutics, Faculty of Pharmacy, Nahda University, Beni-Suef, 62521 Egypt; 5 Biotechnology and Life Science Department, Faculty of Postgraduate Studies for Advanced Sciences, Beni-Suef University, Beni-Suef, Egypt; 6 Department of Pharmacology, Faculty of Veterinary Medicine, Beni-Suef University, Beni-Suef, Egypt

**Keywords:** Atherosclerosis, Diabetes mellitus, Candesartan, Invasomes, Permeation, Toxicity

## Abstract

Atherosclerosis is an inflammatory disease characterized by the accumulation of arterial plaque. Diabetes mellitus stands out as a major risk factor for atherosclerosis. Candesartan is a potent angiotensin II receptor antagonist that enhances arterial blood flow and reduces insulin resistance. However, oral candesartan has limited activity because of its low bioavailability, water solubility, hepatic first-pass degradation, and efficacy. The current study aims to develop nasal candesartan-loaded invasome (CLI) drops to improve candesartan’s permeation, release, and bioavailability as a potential treatment for diabetes-associated atherosclerosis. Design expert software was used to prepare various CLI formulations to determine the impact of the concentrations of ethanol, cineole, and phospholipid. The desirability index was used to estimate the optimized formulation composition to maximize entrapment efficiency and minimize vesicle size. The optimized formulation had a 1% ethanol concentration, a 1.5% cineole concentration, and a 2.32% phospholipid concentration. The selected optimized formulation was then tested in a rat model of diabetes and atherosclerosis to evaluate its activity. The results showed that nasal CLI drops significantly raised serum HDL levels by a ratio of 1.42 and lowered serum glucose, cholesterol, triglycerides, LDL, and VLDL levels by 69.70%, 72.22%, 36.52%, 58.0%, and 65.31%, respectively, compared with diabetic atherosclerotic rats, throwing an insight on the potential for promising anti-diabetic and anti-atherosclerotic activities. Additionally, atherosclerotic lesions were improved in rats treated with CLI, as shown in histopathology. In conclusion, the results of this investigation showed that treatment with nasal CSN-loaded invasome formulation drops prevented the initiation and progression of diabetes-associated atherosclerosis.

**Graphical Abstract:**

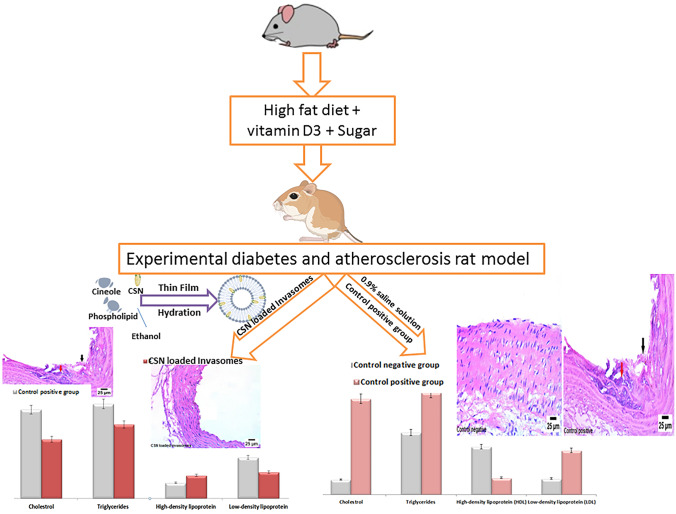

## Introduction

Atherosclerosis is an inflammatory disease in which the intimal layer of the arteries becomes thickened due to hyperlipidemia and lipid oxidation [1, 2]. It is associated with a high risk of cardiovascular death and worldwide mortality [3, 4]. Among the many risk factors for atherosclerosis, diabetes mellitus stands out as a key contributor due to the hyperglycemia that accompanies it and leads to endothelial dysfunction, dyslipidemia, and insulin resistance [3, 5–8]. Numerous prevention therapies, such as high-intensity statins, angiotensin II receptor blockers, and antihyperglycemic agents, have been established for the management of atherosclerosis in diabetic patients because of their anti-inflammatory, antioxidant, and anti-apoptotic properties [9–13]. Several investigations have shown that angiotensin II receptor antagonists are effective anti-atherosclerotic drugs because they inhibit lipid oxidation, restore normal oxidase activity, and boost endothelial function [14–18]. They also reduce hyperglycemia by increasing the body’s sensitivity to insulin [14–18]. It has been estimated that between 53.1 and 72.0% of people with diabetes and atherosclerosis take angiotensin II receptor blockers [9–11]. Candesartan (CSN) is a potent angiotensin II receptor antagonist used to lower the risk of cardiovascular mortality [14, 16, 17]. CSN is an effective vasodilator that increases blood flow on the arterial wall by decreasing NF-κβ, oxidative stress, plaque disruption, and macrophage formation in the aortas [14, 16, 17]. In addition, CSN inhibits insulin resistance and glucose intolerance, leading to a decrease in hyperglycemia [19]. Thus, the purpose of this study was to examine the anti-diabetes-associated atherosclerosis activity of CSN in experimentally induced diabetic atherosclerosis in rats.

Oral CSN has significant limitations due to its limited systemic bioavailability and efficacy [20, 21]. Based on the biopharmaceutics classification system, CSN is categorized as a class II drug because of its poor water solubility (2.04 × 10^−6^ g/mL) and insufficient intestinal absorption caused by its hepatic first-pass degradation [20, 21]. Nasal administration is seen as a viable strategy for drug delivery because of the nasal cavity’s enormous surface area, highly vascularized mucosa, and network of blood vessels that enter directly into the systemic circulation without being subjected to first-pass metabolism [22–24]. While the nasal route has promise, absorption is restricted by the epithelial barrier and mucociliary clearance [22–24]. Invasomes are lipid vesicles composed of phospholipids, cholesterol, terpenes, and ethanol [25–27]. Phospholipids are the lipid particles that form invasomes [27, 28]. Cholesterol was found to increase membrane integrity and drug retention by filling the spaces left behind by the poor packing of other lipid species in vesicular membranes [27, 28]. Terpenes and ethanol both soften the lipid bilayer of the nasal barrier, making it less rigid and increasing its flexibility, diffusion, and fluidity [29, 30]. They improve drug delivery through the nasal mucosa by increasing their penetration capacities [29, 30]. Ethanol also provides a net negative surface charge and limits vesicle aggregation due to electrostatic repulsion, which increases the storage stability of invasomes [27, 29]. Invasomes are flexible nanovesicles used to deliver drugs to their target cells [25, 27]. Invasomes can improve the drug’s bioavailability and effectiveness by increasing the drug’s solubility, release, and permeation through cellular membranes [25–27]. Additionally, invasomes can increase nasal medication residence time due to their phospholipid content, which tends to form thick bilayers and increases the viscosity of invasomal dispersion [31, 32]. Several studies have assessed the transdermal potential of nanoinvasomes containing anti-hypertensive medicines like olmesartan, valsartan, and isradipine, and the nano formulations have shown improved drug flux and permeation, which in turn improves their bioavailability and efficacy [33–35]. However, to our knowledge, there have been no studies that prove CSN-loaded invasomes have improved the release, permeability, or efficacy of CSN. Thus, the purpose of this study was to develop nasal CSN-loaded invasome formulation drops as a potential treatment of diabetes-associated atherosclerosis in experimental rats. CSN-loaded invasome formulation was developed to improve nasal retention time, permeation, and release of CSN, and as a consequence, bioavailability and anti-atherosclerosis activity of CSN can be increased. Design expert software was used to optimize various CSN-loaded invasome formulations using Box-Behnken design. Response surface models like the Box-Behnken design are useful for studying the interplay between several factors with constrained experimental runs [36, 37]. The selected optimized formulation was then evaluated in vivo to examine its anti-atherosclerosis activity in the rat model of experimental diabetes and atherosclerosis.

## Materials and methods

### Materials

AstraZeneca Pharmaceuticals, based in Cairo, Egypt, generously supplied candesartan for this study. We ordered our cholesterol, phospholipids, and cineole from Sigma-Aldrich (St. Louis, MO, USA). We ordered our methanol, ethanol, and chloroform from Cornell Lab (Cairo, Egypt).

### Preparation of plain-unloaded invasomes

The thin-film hydration method was used to develop a plain-unloaded invasome formulation [27]. A solution of chloroform and methanol was used as organic solvent to dissolve phospholipid, cineole, and cholesterol (0.16%), which then evaporated using a rotary evaporator (Rotavapor, Heidolph VV 2000, Burladingen, Germany) under vacuum at 40 °C and 100 rpm to form a thin film of plain invasomes. A solution of phosphate buffer (pH 6.8) containing ethanol was used to rehydrate the plain invasomal film at 60 rpm, and the resulting suspension was kept at 4 °C.

### Experimental design

To determine the impact of the concentrations of ethanol (*X*_1_), cineole (*X*_2_), and phospholipid (*X*_3_), a 3^3^ Box-Behnken design was generated in Design-Expert software^®^ (version 12.0.6.0, StatEase Inc., Minneapolis, MN, USA) to prepare 15 formulations of CSN-loaded invasomes as shown in Tables 1 and 2. The Box-Behnken design is a common choice for optimizing invasomes’ parameters, where a small number of process variables can have a significant impact on product quality [33, 38]. It has the added benefit of not requiring you to consider any permutations in which every single consideration is at its most extreme value. This makes these plans useful for avoiding extreme experiments when undesirable results are possible [33, 38]. Entrapment efficiency (EE%) and vesicle size were measured as dependent variables.

**Table 1:** The independent and dependent variables of CSN-loaded invasomes

**Independent variables**	**Coded values**					
	** − 1**			0		** − 1**
*X*1: concentration of ethanol (%)	1			3		5
*X*2: concentration of cineole (%)	0.5			1		1.5
*X*_3_: concentration of phospholipid (%)	1			3		5

**Dependent variables**	**Model**	**Lack-of-fit**	**df**	***F*-Value**	***p*-value**	**Constraints**
*Y*_1_: entrapment efficiency (%)	Quadratic	0.9457	9	1110.88	< 0.0001	Maximize
*Y*_2_: vesicle size (nm)	Quadratic	0.9984	9	839.47	< 0.0001	Minimize

*df* degrees of freedom which is the number of estimated parameters required to compute the sum of squares of the source

**Table 2:** Compositions and responses of CSN-loaded invasomes

**Run**	**Factors levels in actual values**			**Responses (*n*** **= 3)**	
	***X***_**1**_**: concentration of ethanol (%)**	***X***_**2**_**: concentration of cineole (%)**	***X***_**3**_**: concentration of phospholipid (%)**	**Entrapment efficiency (% ± SD)**	**Vesicle size (nm ± SD)**
**F1**	5	1	5	86.67 ± 0.34	297.70 ± 1.87
**F2**	5	1	1	72.54 ± 0.36	172.43 ± 3.97
**F3**	3	1	3	80.31 ± 0.29	228.23 ± 4.31
**F4**	3	1	3	80.48 ± 0.48	228.73 ± 5.45
**F5**	3	1	3	80.51 ± 0.47	229.17 ± 3.74
**F6**	3	0.5	5	84.74 ± 0.33	283.87 ± 3.76
**F7**	3	1.5	1	76.44 ± 0.44	202.60 ± 2.95
**F8**	1	1	1	74.68 ± 0.47	188.57 ± 2.82
**F9**	1	1	5	88.59 ± 0.32	313.53 ± 5.73
**F10**	1	0.5	3	78.58 ± 0.37	213.77 ± 4.65
**F11**	3	1.5	5	90.29 ± 0.37	327.67 ± 2.23
**F12**	5	1.5	3	82.47 ± 0.54	242.43 ± 4.33
**F13**	3	0.5	1	70.64 ± 0.45	158.70 ± 3.99
**F14**	1	1.5	3	84.44 ± 0.34	257.70 ± 6.34
**F15**	5	0.5	3	76.63 ± 0.33	198.07 ± 2.46

*SD* standard deviation

### Preparation of CSN-loaded invasomes

The thin-film hydration method was used to develop 15-CSN-loaded invasome formulations [27]. A solution of chloroform and methanol was used as an organic solvent to dissolve CSN (10 mg), phospholipid, cineole, and cholesterol (0.16%) which then evaporated using a rotary evaporator under vacuum at 40 °C and 100 rpm to form a thin film of invasomes. A solution of phosphate buffer containing ethanol was used to rehydrate the invasomal film at 60 rpm, and the resulting suspension was kept at 4 °C.

### Characterization of CSN-loaded invasomes

#### Entrapment efficiency measurement

In order to separate un-entrapped CSN from CSN-loaded invasomes, a cooling centrifuge (SIGMA 3–30 K, Sigma, Steinheim, Germany) was used at 15,000 rpm for 1 h. The concentration of un-entrapped CSN was determined using a UV–visible spectrophotometer at a wavelength of 258 nm based on the standard calibration curve. The following equation was used to determine EE% after triplicate measurements [39]:

1 $$\mathrm{Entrapment}\;\mathrm{efficiency}\;(\%)=(({\mathrm C}_{\mathrm t}-{\mathrm C}_{\mathrm f})/{\mathrm C}_{\mathrm t})\times100$$

where *C*_t_ is the total amount of CSN and *C*_f_ is the amount of free CSN.

#### Vesicle size measurement

A sample of different CSN-loaded invasome formulations was diluted and assessed for measuring the vesicle size in triplicates by dynamic light scattering using a Zetasizer instrument (Malvern, Worcestershire, UK) [40].

### Optimization of CSN-loaded invasomes

Each dependent variable’s results was analyzed using analysis of variance (ANOVA) in Design-Expert^®^ software, with criteria including lack of fit, *F*-value, degree of freedom, and *p*-value used to determine which model was the best fit for the data [41]. Optimization based on the desirability index was achieved by imposing the constraints of the largest entrapment efficiency and the smallest vesicle size. The software’s computed optimized formulation factors and projected responses were then verified by preparing and evaluating the indicated optimized formulation in triplicate.

### Characterization of the optimized CSN-loaded invasome formulation

#### Differential scanning calorimetry (DSC)

To investigate the interaction between CSN and the invasomal producing components, the thermograms of CSN, cholesterol, phospholipid, and optimized CSN-loaded invasomes were evaluated using a Shimadzu DSC-50 differential scanning calorimeter (Shimadzu Corporation, Kyoto, Japan) [28]. At a steady 5 °C/min, the DSC scan was acquired over a 25–250 °C temperature range with a constant flow of 100 mL/min of nitrogen.

#### Fourier transform infrared spectroscopy (FT-IR)

The chemical interactions and crystallinity index of CSN, cholesterol, phospholipid, and optimized CSN-loaded invasomes were observed by FTIR (8400s, Shimadzu, Japan) [42]. The samples were thoroughly pulverized and mixed with KBr before being analyzed from 4000 to 400 cm^−1^.

#### Transmission electron microscopy (TEM)

The optimized CSN-loaded invasome formulation was characterized morphologically using TEM (JEM-1230, Jeol, Tokyo, Japan) [43]. A sample of the CSN-loaded invasome formulation was diluted and dyed with 2% phosphotungstic acid after being placed on a carbon-coated grid.

#### Zeta potential and size distribution

Zeta potential is a deterministic indicator of the strength of repulsive and attractive forces between vesicles, allowing for an evaluation of the invasomes’ stability [40]. In order to measure the distribution and vesicle size homogeneity within a sample, the polydispersity index (PDI) was used [40]. A sample of optimized CSN-loaded invasome formulation was diluted and assessed for measuring the zeta potential and PDI in triplicates by dynamic light scattering using a Zetasizer instrument.

#### In vitro release and kinetics studies

By employing a diffusion technique based on cellophane dialysis bags (molecular weight cut off 12,000; Sigma-Aldrich, Cairo, Egypt), we compared the drug release from the optimized CSN-loaded invasomes to that from free CSN suspension [41]. Dialysis bags were filled with a CSN-loaded invasome formulation and a free CSN suspension (equal to 3 mg CSN). The bags were then placed in a Hanson dissolution apparatus (Hilab, Düsseldorf, Germany) with 50 mL of phosphate-buffered saline (pH 6.8) containing 0.1% (w/w) Tween 80 as a medium of release that was greater than the saturation solubility of CSN to ensure the sink condition at 37 °C and 100 rpm. Three-milliliter samples were taken at regular intervals over 24 h and replaced with an equivalent volume of new dissolving media to maintain the sink condition. The concentration of CSN in each sample was determined using a UV–visible spectrophotometer calibrated against a standard curve at a wavelength of 258 nm. The results of CSN cumulative release percentage were replicated three times as mean ± SD.

The release kinetics study was calculated by applying the collected data to the models found in the DDSolver program. The values of the Akaike information criterion (AIC), model selection criterion (MSC), and correlation coefficient (*R*^2^) were compared to determine the best-fitting release model [40]. The Korsmeyer-Peppas model was then used to examine the CSN release mechanism from the optimized CSN-loaded invasome formulation.

#### Ex vivo permeation study

Ex vivo permeation of free CSN and the optimized CSN-loaded invasome formulation were studied using epidermal layer of abdominal skin excisions from albino rats as diffusion membranes. The ethics committee at Beni-Suef University in Egypt gave its stamp of approval to the rat skin collection and processing. In this experiment, we employed a Hanson dissolution apparatus at 37 °C and 100 rpm and a receptor medium of 50 mL of phosphate-buffered saline (pH 6.8) containing 0.1% (w/w) Tween 80 [27]. Three mg of CSN in both the CSN-loaded invasome formulation and the free CSN suspension was evaluated. Three-milliliter samples were taken at regular intervals over 24 h and replaced with an equivalent volume of new dissolving media to maintain the sink condition. The concentration of CSN in each sample was determined using a UV–visible spectrophotometer calibrated against a standard curve at a wavelength of 258 nm. The results of CSN cumulative permeation percentage and steady state flux were replicated three times as mean ± SD.

### In vivo evaluation of optimized CSN-loaded invasomes

#### Animals

In an air-conditioned room with a humidity of 30–70%, 30 adult male albino rats (200–250 g body weight) were kept with free access to food and drink. The rats were given an adapted diet for a week, then weighed and separated into five groups of six (*n* = 6). All groups’ starting weights, triglycerides (TG), high-density lipoprotein (HDL), low-density lipoprotein (LDL), very LDL (VLDL), cholesterol (C), red blood cells (RBCs) count, total white blood cells (WBCs) count, blood glucose, serum alanine aminotransferase (ALT), serum aspartate aminotransferase (AST), serum urea, and serum creatinine were recorded before the trial began. The ethics committee at Beni-Suef University in Egypt gave their stamp of approval (BSU-IACUC: 022–391) to the rat skin collection and processing.

#### Treatment protocol

Group 1 (*n* = 6) acted as a control negative group and received an intraperitoneal injection of 0.9% saline solution with a normal diet throughout the study. To induce atherosclerosis, rats (*n* = 24) were given two intraperitoneal injections of vitamin D3 (600,000 IU/kg) with a high-fat diet of 6% cholesterol, 1% sodium cholate, 0.2% propyl thiouracil, and 5% refined sugar for 12 weeks [44]. Five percent refined sugar was added to induce diabetes in rats and consequently hasten the induction of atherosclerosis [44]. Measurement of glucose level was used to confirm the successful induction of diabetes [45]. After induction of atherosclerosis, rats (*n* = 24) were randomly divided into four groups of six. The second group acted as a positive control and received 0.9% saline solution via gavage on a daily basis. The third group received free CSN (10 mg/kg) via oral gavage on a daily basis [46] The fourth group received plain invasomes via the nasal route. The fifth group received CSN-loaded invasomes (10 mg/kg) via the nasal route. The rats were weighed every week and observed for any change in behavior throughout the study period [47]. Rats were given an intraperitoneal injection of a mixture of ketamine (90 mg/kg) and xylazine (5 mg/kg) containing a 0.1 mg/100 gm concentration before being terminated by cervical dislocation at the end of the research.

#### Anti-atherosclerosis activity measurement

Serum was separated from blood drawn from the abdominal aortic artery and investigated for C, TG, LDL, HDL, and VLDL levels as per the instructions provided by the manufacturer [47, 48].

Samples of thoracic aortas were fixed in 10% neutral buffer formalin, then dehydrated in ethyl alcohol, cleaned in xylene, and embedded in paraffin. Hematoxylin and eosin staining were performed on these samples for histopathological analysis [48].

#### Toxicity studies

Every rat’s weight was recorded before, throughout, and on the day of sacrifice [49]. Death, aging, and other visible changes in appearance and attitude were also seen [49].

Serum was separated from blood drawn from the abdominal aortic artery and investigated for hematological parameters, like hemoglobin (Hb), RBC, MCV (mean corpuscular volume), mean corpuscular hemoglobin (MCH), mean corpuscular hemoglobin concentration (MCHC), WBCs, differential WBCs (neutrophil, lymphocyte, and monocyte), and platelet count [49].

Serum was separated and investigated for creatinine, urea, AST, ALT, and blood glucose [49].

### Statistical analysis

The data was evaluated statistically using SPSS (version 22.0. Chicago, USA), with all values reported as the mean ± SD (standard deviation) and the findings assessed using one-way ANOVA and Tukey’s post hoc test at a *p*-value < 0.05.

## Results

### Experimental design

In this investigation, successful preparation and optimization of CSN-loaded invasomes were achieved using the Box-Behnken design. As can be seen in Table 1, the quadratic model was chosen for EE% and vesicle size. This model was selected because it has an insignificant lack-of-fit and a significant *p*-value and *F*-value. Additionally, the model can be used to interpolate EE% and vesicle size data with high confidence due to its predicted *R*^2^ of 0.9942 and 0.9924, respectively, and adjusted *R*^2^ of 0.9956 and 0.9942, respectively. With an adequate precision of 112.066 and 94.237 for EE% and vesicle size, respectively, the model has sufficient signal for optimization and can be used to confirm the design process.

### Characterization of CSN-loaded invasomes

#### Entrapment efficiency

**Fig. 1:**
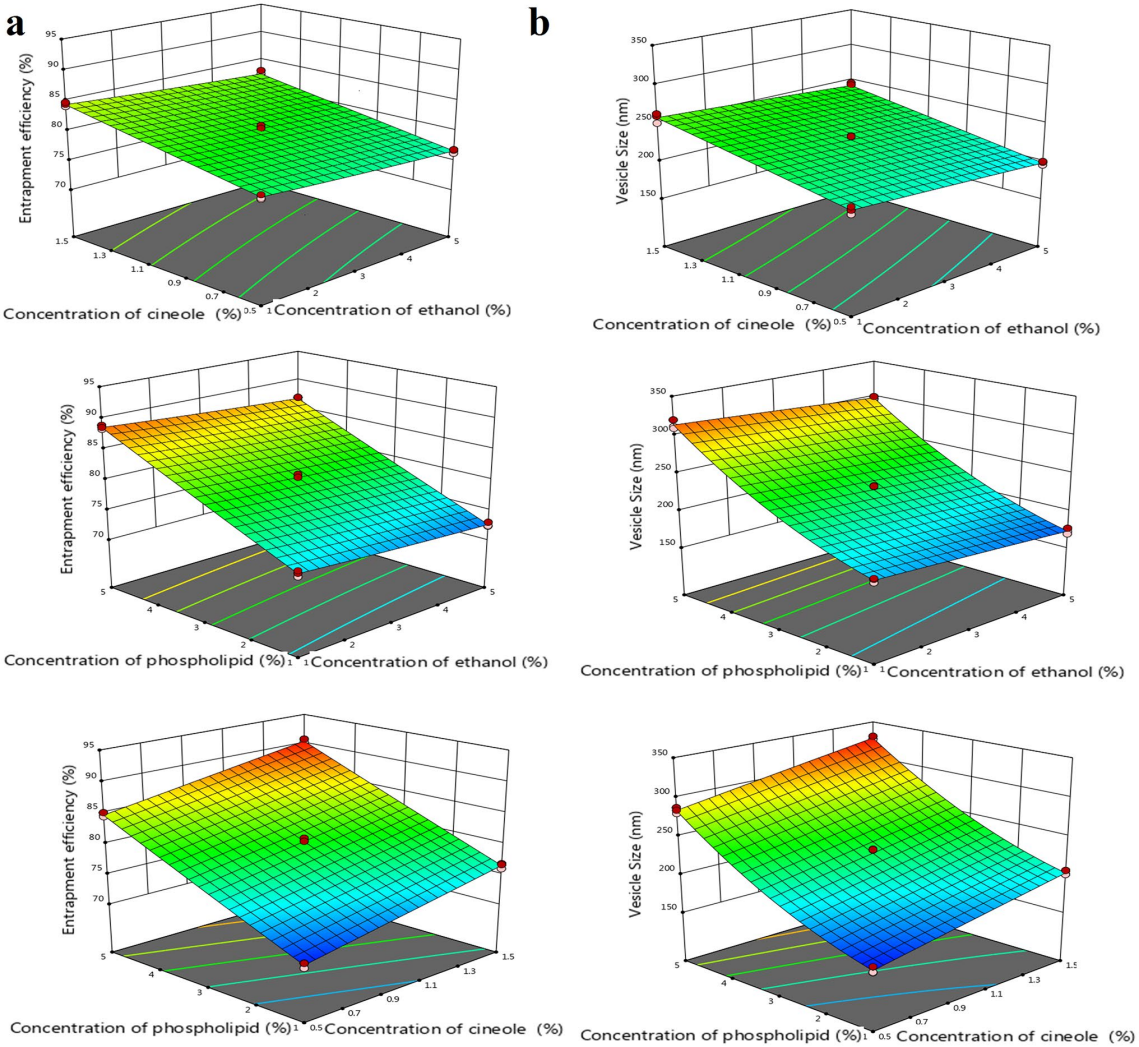
3D response surface plot for the effect of concentration of ethanol (*X*_1_), concentration of cineole (*X*_2_), and concentration of phospholipid (*X*_3_) on EE% (**a**) and vesicle size (**b**)

According to Table 2, the EE% of the CSN-loaded invasome formulations varied from 70.64 to 90.29%. Figure 1 and Eq. 2 reveal that all three independent variables had a statistically significant (*p*-value < 0.05) effect on the EE%. Ethanol (*X*_1_) has a negative impact on EE%, where EE% of F1 (formulation with 5% ethanol) and F9 (formulation with 1% ethanol) was 86.67 ± 0.34% and 88.59 ± 0.32%, respectively. Cineole (*X*_2_) has a positive impact on EE%, where EE% of F6 (formulation with 0.5% cineole) and F11 (formulation with 1.5% cineole) was 84.74 ± 0.33% and 90.29 ± 0.37%, respectively. Phospholipid (*X*_3_) has a positive impact on EE%, where EE% of F2 (formulation with 1% phospholipid) and F1 (formulation with 5% phospholipid) was 72.54 ± 0.36% and 86.67 ± 0.34%, respectively.

2 $$\begin{aligned}\mathrm{Entrapment}\;\mathrm{efficiency}\;(\mathrm{EE}\%)=&+\;80.53-0.9950 X_1+2.88 X_2\\&+7.00X_3-0.0064 X_1 X_2\\&+0.0553 X_1 X_3-0.0624 X_2 X_3\\&+0.0945 X_1{^2}+0.0039\mathrm X_2{^2}\\&+0.0931X_3{^2}\end{aligned}$$

#### Vesicle size

According to Table 2, the vesicle size of the CSN-loaded invasome formulations varied from 158.7 to 327.67 nm. Figure 1 and Eq. 3 reveal that all three independent variables had a statistically significant (*p*-value < 0.05) effect on the vesicle size. Ethanol (*X*_1_) has a negative impact on vesicle size, where vesicle size of F12 (formulation with 5% ethanol) and F14 (formulation with 1% ethanol) was 242.43 ± 4.33 nm and 257.70 ± 6.34 nm, respectively. Cineole (*X*_2_) has a positive impact on vesicle size, where vesicle size of F10 (formulation with 0.5% cineole) and F14 (formulation with 1.5% cineole) was 213.77 ± 4.65 nm and 257.70 ± 6.34 nm, respectively. Phospholipid (*X*_3_) has a positive impact on vesicle size, where vesicle size of F7 (formulation with 1% phospholipid) and F11 (formulation with 5% phospholipid) was 202.60 ± 2.95 nm and 327.67 ± 2.23 nm, respectively.

3 $$\begin{aligned}\mathrm{Vesicle\;size}=&+ \;228.71-7.87X_1+22.00X_2+62.56X_3\\&+0.1083X_1X_2+0.0750X_1X_3-0.0250X_2X_3\\&-0.4347X_1{^2}-0.2847X_2{^2}+14.78X_3{^2}\end{aligned}$$

### Optimization of CSN-loaded invasome formulations

**Fig. 2:**
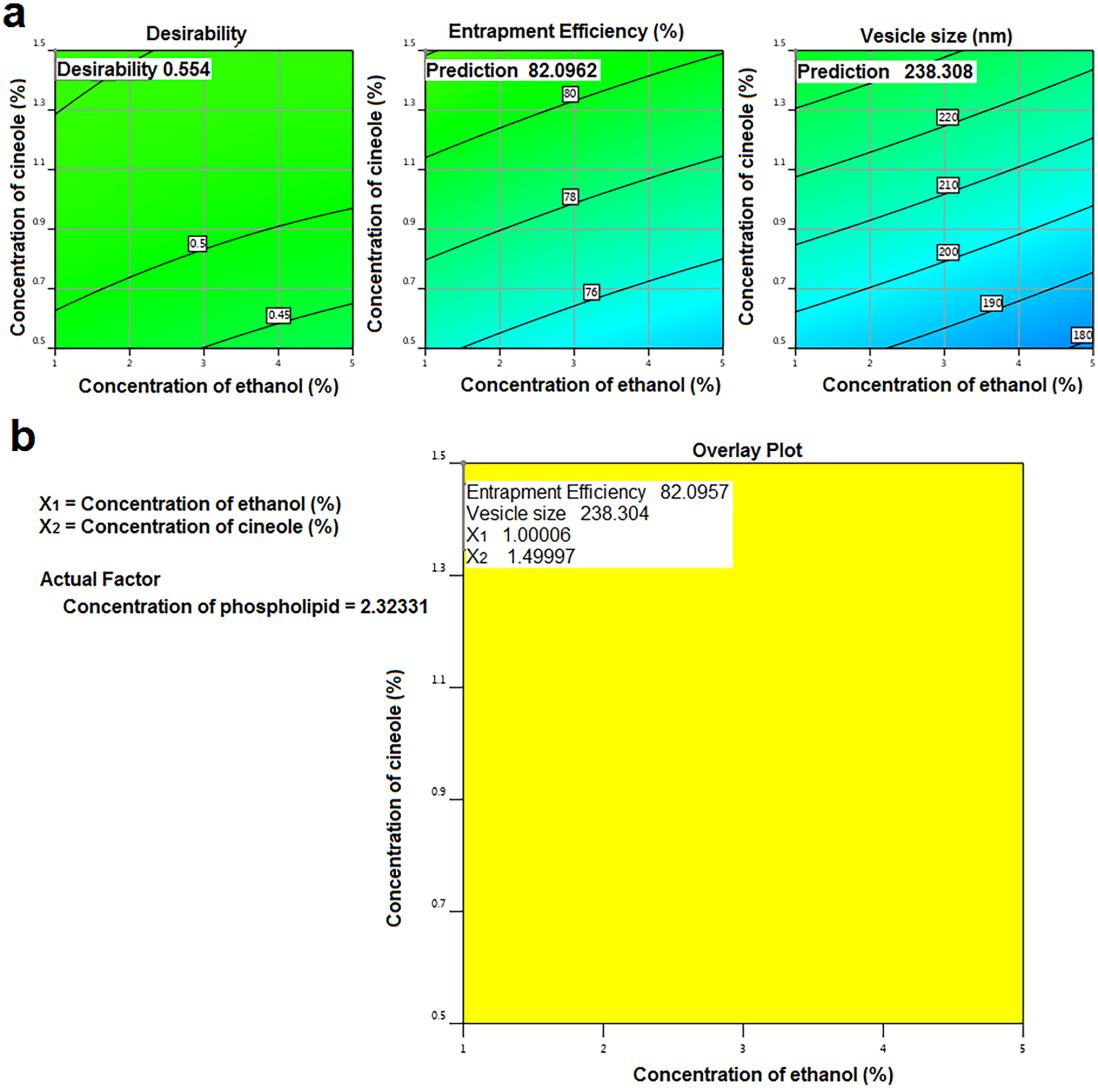
The contour (**a**) and overlay response surface (**b**) plots representing the predicted and observed values for the optimized CSN-loaded invasomes

A recommended optimized formulation was proposed by the design expert program (Table 1) after vesicle size and EE% constraints were applied. Figure 2 shows that the most desirable formulation had a 1% ethanol concentration, a 1.5% cineole concentration, and a 2.32% phospholipid concentration. Predicted values of responses were verified by in vitro studies of the optimized CSN-loaded invasome formulation, with an average EE% of 82.04 ± 0.43% and a vesicle size of 228 ± 3.15 nm.

### Characterization of the optimized CSN-loaded invasomes

#### Differential scanning calorimetry (DSC)

**Fig. 3:**
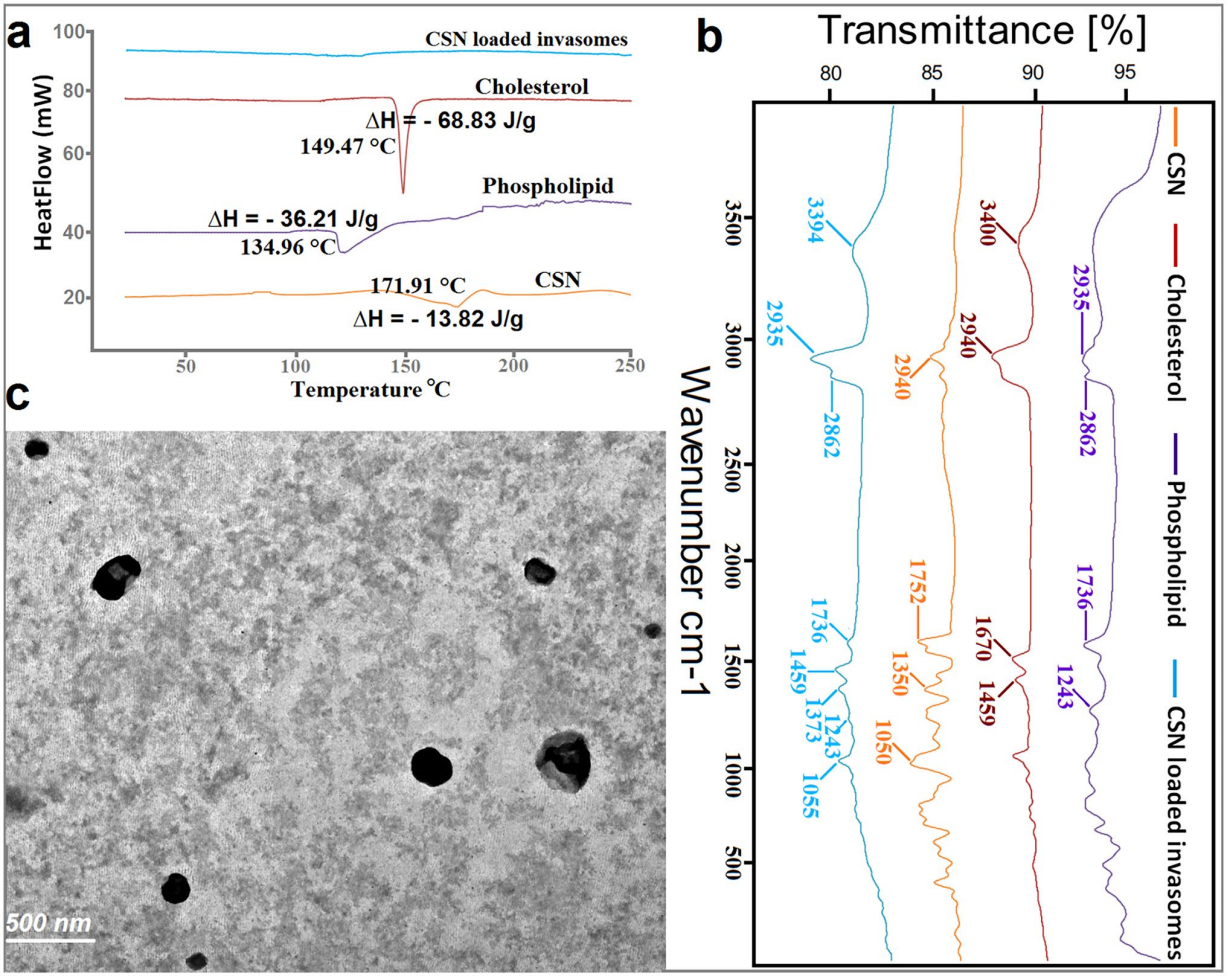
**a** DSC thermograms, **b** FTIR spectrums, and **c** TEM micrograph for the optimized CSN-loaded invasomes. ∆*H* is the enthalpy

As shown in Fig. 3a, the CSN thermogram displayed an endothermic peak at 171.91 °C, which is also its melting point. The phospholipid thermogram displayed a sharp endothermic peak at 134.96 °C, which is also its melting point. The cholesterol thermogram displayed an endothermic peak at 149.47 °C, which is also its melting point. CSN-loaded invasome thermogram displayed the disappearance of these peaks.

#### FTIR

Figure 3b displays the FTIR spectrums of CSN, phospholipid, cholesterol, and an optimized CSN-loaded invasomes. The phospholipid FTIR spectrum showed peaks at 2935 and 2862 cm^−1^ for the CH_2_ stretching vibration, at 1736 cm^−1^ for the symmetrical C = O stretching vibration, and at 1243 cm^−1^ for the PO_4_ antisymmetric stretching bands. The cholesterol FTIR spectrum showed peaks at 3400 cm^−1^ for the OH stretching group, at 2940 cm^−1^ for the CH_2_ stretching vibration, at 1670 cm^−1^ for the double bond C = C, and at 1459 cm^−1^ for the asymmetric CH_2_ stretching vibrations. The CSN FTIR spectrum showed peaks at 2940 cm^−1^ for the CH stretching group, at 1752 cm^−1^ for the carbonyl C = O stretching, at 1350 cm^−1^ for the aromatic C–N stretching, and at 1080 cm^−1^ for the C–O ether stretch. Similar peaks were observed in the FTIR spectrum of the optimized CSN-loaded invasomes, indicating that there was compatibility between components of the formulation.

#### Transmission electron microscopy (TEM)

Micrographs taken with a TEM (Fig. 3c) showed the formation of spherical vesicles without aggregates and with a clear outline and core.

#### Zeta potential and size distribution

The PDI for the optimized formulation was 0.220, proving that uniform vesicles were developed. The zeta potential for the optimized formulation was − 35.8 mV, which is indicative of increased vesicles stability and reduced aggregation tendency.

#### In vitro release studies

**Fig. 4:**
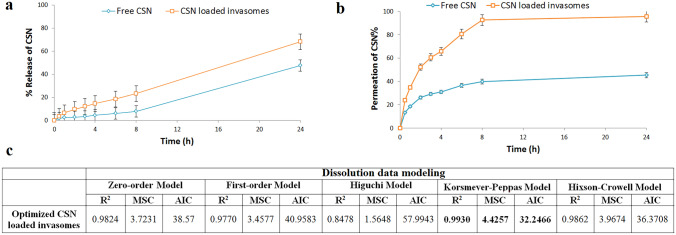
**a** In vitro release profile, **b** ex vivo drug permeation profile, and **c** release kinetics from the optimized CSN-loaded invasomes. AIC Akaike information criterion, MSC model selection criterion, and *R*^2^ correlation coefficient

Figure 4a shows the in vitro drug release profile of CSN-loaded invasomes compared to free CSN dispersion. Using the Student *t* test, we found that the total amount of CSN released from the CSN-loaded invasomes was significantly (*p*-value < 0.05) higher than the total amount of CSN released from the free CSN dispersion, with an enhancement ratio of 1.43.

Figure 4c displays the results of a DDSolver calculation of the kinetic release mechanism of CSN from the optimized CSN-loaded invasomes. The optimized CSN-loaded invasomes had the best fit with the Korsmeyer-Peppas model because it had the highest MSC and *R*^2^ and the lowest AIC, with *n* value of 0.871, indicating a non-Fickian diffusion release mechanism.

#### Ex vivo skin permeation study

Figure 4b shows the ex vivo drug permeation profile from CSN-loaded invasomes compared to free CSN dispersion. Using the Student *t* test, we found that the total amount of CSN permeated from the free CSN dispersion (272.59 ± 4.77) was significantly (*p*-value < 0.05) less than the total amount of CSN permeated from the CSN-loaded invasomes (382.99 ± 2.01 µg/cm^2^). Additionally, the flux (Jss) of CSN-loaded invasomes (26.73 ± 0.27 µg/cm^2^/h) was significantly (*p*-value < 0.05) higher than that of free CSN (13.77 ± 0.08 µg/cm^2^/h) with an enhancement ratio of 1.94.

### In vivo evaluation of CSN-loaded invasomes

#### Anti-atherosclerosis activity measurement

**Fig. 5:**
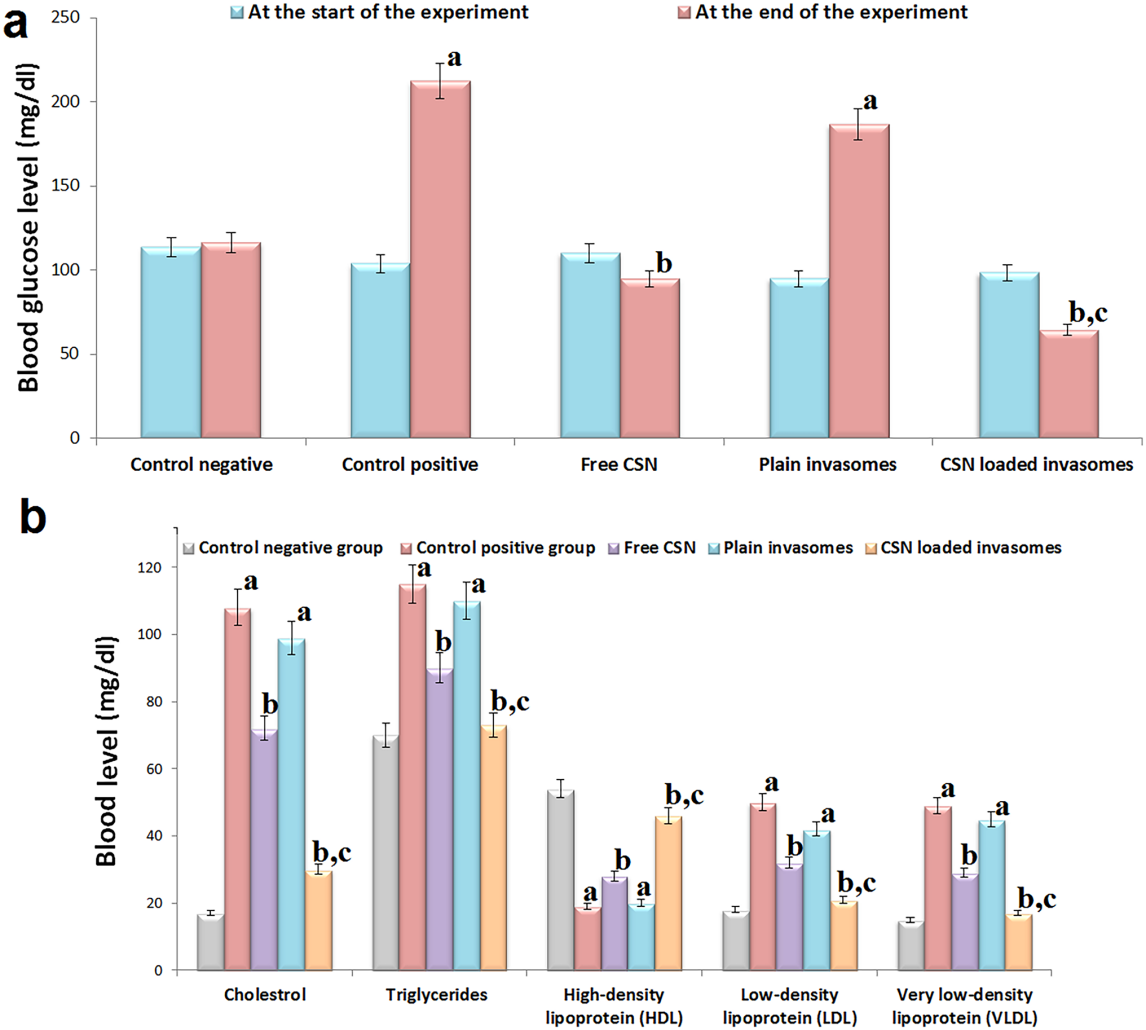
The changes of blood **a** glucose level and **b** lipid profile of rats among different treatment groups. Significance: ^a^*p*-value < 0.05 versus the control negative group; ^b^*p*-value < 0.05 versus the control positive group; ^c^*p*-value < 0.05 versus the free CSN group

Figure 5a demonstrates that inducing diabetes in rats was successful because the blood glucose level in the control positive group was significantly (*p*-value < 0.05) higher than that of the control negative group. Additionally, Fig. 5a demonstrates that both free CSN and CSN-loaded invasomes were found to have anti-diabetic activity because their blood glucose levels were significantly (*p*-value < 0.05) lower than those of the control positive group. Figure 5b demonstrates that inducing atherosclerosis in rats was successful because serum C, TG, LDL, and VLDL levels were significantly (*p*-value < 0.05) higher, and HDL was significantly (*p*-value < 0.05) lower than those of the control negative group. Additionally, Fig. 5b demonstrates that both free CSN and CSN-loaded invasomes were found to have anti-atherosclerotic activity because their serum C, TG, LDL, and VLDL levels were significantly (*p*-value < 0.05) lower, and HDL was significantly (*p*-value < 0.05) higher than those of the control positive group. Among treatment groups, the CSN-loaded invasome group had the lowest serum C, TG, LDL, and VLDL levels with a decrease of 72.22%, 36.52%, 58.0%, and 65.31%, respectively, and the highest serum HDL level with an increased ratio of 1.42. Compared to the free CSN group, the CSN-loaded invasome group showed a statistically significant (*p*-value < 0.05) increase in HDL by a ratio of 3 and a decrease in serum C, TG, LDL, and VLDL by a ratio of 2.16, 1.68, 1.61, and 1.60, respectively. There was no statistically significant difference (*p*-value > 0.05) between the blood glucose, C, TG, LDL, VLDL, and HDL levels of the control positive group and the plain invasome group.

**Fig. 6:**
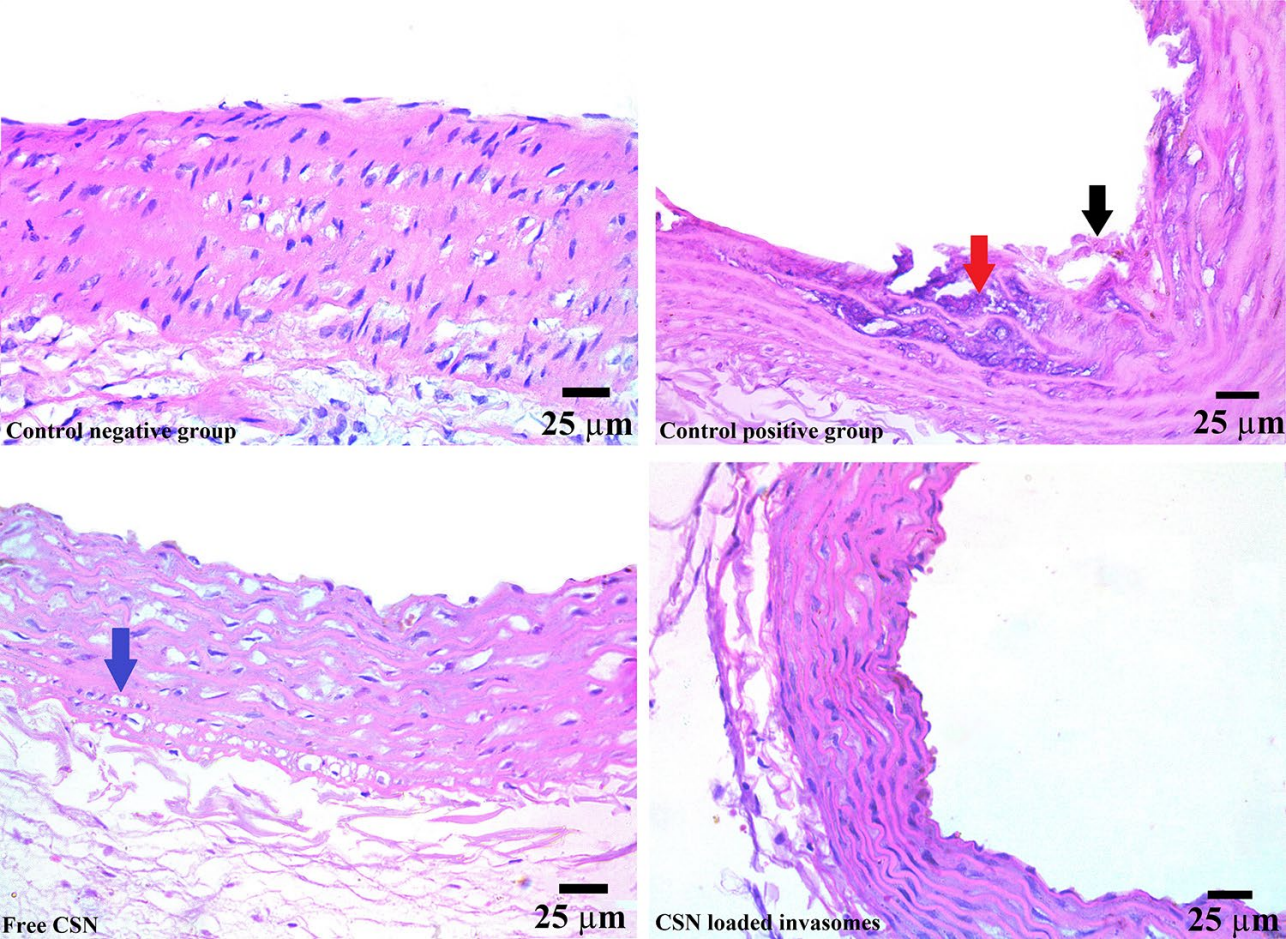
Histopathology images of aortic wall of rats among different treatment groups. The images are representative for atherosclerosis (control positive group) in addition to atherosclerosis-treated groups (free SCN, CSN-loaded invasomes)

Figure 6 confirms the induction of atherosclerosis in rats. While the aortic wall appeared normal in the photomicrographs of the control negative group, the aortic wall of the control positive group was thickened due to the formation of atherosclerotic plaques and the deposition of a high amount of calcium (red arrow), accompanied by severe destruction in the tunica intima and tunica media (black arrow). Foam cell formation and exfoliation of the vascular endothelium were also visible in photomicrographs from the control positive group. Additionally, Fig. 6 demonstrates that atherosclerotic lesions in both free CSN and CSN-loaded invasome groups were improved, indicating their anti-atherosclerotic activity. Among treatment groups, the CSN-loaded invasome group had the highest activity with a relatively intact aorta intimal structure. Although atherosclerotic lesions improved in the free CSN group, there was local thickening of the aorta wall and a moderate infiltration of foam cells (blue arrow) in the tunica media.

#### Toxicity studies

No signs of toxicity or mortality were observed in rats after daily nasal administration of CSN-loaded invasomes, with no abnormalities in behavior when compared with the control positive group. Measurement of body weight demonstrated that the body weight of CSN-loaded invasomes was insignificantly (*p*-value > 0.05) increased compared with the control negative group, indicating normal growth. Table 3 shows that, as compared to the negative control group, the hematological and biochemical parameters were not significantly (*p*-value > 0.05) different after nasal delivery of CSN-loaded invasomes, indicating no toxicity concerns.

**Table 3:** Hematological and biochemical parameters of CSN-loaded invasomes

**Type**	**Parameters**	**Unit**	**Control negative group**	**CSN-loaded invasomes**
	Hemoglobin	g/L	13.1 ± 0.85	13.4 ± 2.0^a^
	Red blood cells	100^3^/µL	7.2 ± 0.56	7.76 ± 0.93^a^
	Hematocrit	%	39.88 ± 1.84	40.5 ± 4.44^a^
	Mean corpuscular volume	fL/cell	52.36 ± 2.56	52.83 ± 1.1^a^
	Mean corpuscular hemoglobin	pg/cell	16.86 ± 0.81	16.9 ± 0.36^a^
**Hematological**	Mean corpuscular hemoglobin concentration	g/dL	31.67 ± 1.52	32.0 ± 1.0^a^
	Platelet count	10^3^/mm^3^	538.33 ± 16.07	545.5 ± 36.06^a^
	Total white blood cells	10^3^/mm^3^	17.5 ± 2.5	18.86 ± 3.71^a^
	Neutrophils	%	18.33 ± 1.52	20.26 ± 5.86^a^
	Lymphocytes	%	76.83 ± 4.25	80.06 ± 4.74^a^
	Monocytes	%	2.1 ± 0.1	2.2 ± 0.26^a^
**Biochemical**	Urea	mg/dL	37.67 ± 6.80	34.67 ± 3.51^a^
	Creatinine	mg/dL	0.46 ± 0.11	0.47 ± 0.15^a^
	Alanine aminotransferase	U/L	45.67 ± 4.04	51.33 ± 8.08^a^
	Aspartate aminotransferase	U/L	135.0 ± 5.0	132.67 ± 2.51^a^

Values are expressed as the mean ± SD, using one-way ANOVA followed by Tukey’s test at *p*-value 0.05

Insignificance: ^a^*p*-value >0.05 versus control negative group

## Discussion

Atherosclerosis is a common disease caused by the accumulation of plaque within the arterial walls [2, 50]. Diabetes mellitus is associated with an increased risk of developing atherosclerosis [3, 5]. The current study aims to develop nasal CSN-loaded invasome drops as a potential treatment of diabetes-associated atherosclerosis in experimental rats. Invasomes are lipid vesicles composed of phospholipids, cholesterol, terpenes, and ethanol [25–27]. Phospholipids are the lipid particles that form invasomes [27, 28]. Cholesterol was found to increase membrane integrity and drug retention by filling the spaces left behind by the poor packing of other lipid species in vesicular membranes [27, 28]. Terpenes and ethanol both soften the lipid bilayer of the nasal barrier, making it less rigid and increasing its flexibility, diffusion, and fluidity [29, 30]. They improve drug delivery through the nasal mucosa by increasing their penetration capacities [29, 30]. Ethanol also provides a net negative surface charge and limits vesicle aggregation due to electrostatic repulsion, which increases the storage stability of invasomes [27, 29]. Previous studies evaluated the safety of invasomes forming ingredients upon application to the targeted organ [29, 51, 52]. Ethanol has been confirmed to be non-irritating and safe for use as a penetration enhancer [52]. Since terpenes are naturally occurring in essential oils, they are generally considered to be safe and non-irritating [29, 51]. Two skin cell lines were used to test the toxicity of terpenes, and the results showed that terpenes are generally believed to be safe based on the MTT assay and examination of transepidermal water loss (TEWL) [51]. Moreover, the safety of CSN-loaded invasomes was assessed through in vivo tests in animals by comparing them to a control group, with outcomes like mortality, hematological changes, body weight variations, and renal and liver malfunction being observed. In our study, different formulations of CSN-loaded invasomes were developed using the Box-Behnken design and characterized for EE% and vesicle size. Following the collection of data, Design-Expert software determined which model best fit the data using the ANOVA test [53]. The program determines different parameters such as *p*-value, lack-of-fit, *F*-value, predicted *R*^2^, adjusted *R*^2^, and adequate precision to evaluate whether or not a particular model adequately explains the data. The *p*-value of the chosen model must be less than 0.05 to be significant. Lack-of-fit is the degree to which a model’s predictions deviate from observations, so the selected model should have an insignificant lack-of-fit [36]. The *F*-value is a test for contrasting the mean square of the source with that of the residuals, so the selected model should have the highest *F*-value. The model is considered reliable if it has the highest values of predicted *R*^2^ and adjusted *R*^2^ with a difference less than 0.2 between them [53]. The model is considered to be of sufficient accuracy for optimization if the adequate precision is greater than 4 [53]. Based on the analyzed data, the quadratic model was chosen for EE% and vesicle size. In order to determine the effect of each variable and interpret the model, the software also provides coded polynomial equations and 3D surface graphs [36, 43].

CSN was successfully encapsulated in the invasomal formulations, with EE% ranging from 70.64 to 90.29%, demonstrating that invasomes can be used as an effective delivery system for CSN. All three independent variables had a statistically significant (*p*-value < 0.05) effect on the EE%. The EE% of CSN-loaded invasome formulations with high ethanol content was lower than that of formulations with low ethanol concentration. This is because ethanol at greater concentrations can dissolve phospholipid and cause the bilayer to become permeable and leaky [25, 29, 54]. This result agrees with those previously published by Jain et al. [25]. The EE% of CSN-loaded invasome formulations with high cineole content was higher than that of formulations with low cineole concentration. This is because cineole at greater concentrations can dissolve lipophilic drugs such as CSN and hence enter the lipid bilayer and increase its EE% [55, 56]. This result agrees with those previously published by Ahmed et al. [55].The EE% of CSN-loaded invasome formulations with high phospholipid content was higher than that of formulations with low phospholipid concentration. This is because phospholipid at greater concentrations can increase the number of lipid particles forming each vesicle, allowing more space for the CSN to integrate within the invasomes [31, 55]. This result agrees with those previously published by Teaima et al. [31]. Evaluating the vesicle size of invasomes is critical for nasal delivery because the smaller the vesicle size of the invasomal formulation, the larger its surface area and the higher its absorption [25, 29, 54]. Results of vesicles size showed that the vesicle size of all CSN-loaded invasome formulations fell within the nanometer range. All three independent variables had a statistically significant (*p*-value < 0.05) effect on the vesicle size. The vesicle size of CSN-loaded invasome formulations with high ethanol content was lower than that of formulations with low ethanol concentration. This is because the ability of ethanol to penetrate hydrocarbon chains changes the vesicles’ net charge in a way that results in steric stabilization and reduces the average vesicle size [25, 29, 54]. This result agrees with those previously published by Jain et al. [25]. The vesicle size of CSN-loaded invasome formulations with high cineole content was higher than that of formulations with low cineole concentration. When high concentration cineole is incorporated into the lipid bilayer of invasomes, the integral packing of the lipid bilayer is disrupted, and more membrane surface area is needed to accommodate the invasomes [55, 56]. This result agrees with those previously published by El-Tokhy et al. [56]. The vesicle size of CSN-loaded invasome formulations with high phospholipid content was higher than that of formulations with low phospholipid concentration. This is because phospholipid at greater concentrations had a propensity to create thick bilayers, which boosted the vesicle size of the invasomes [31]. At higher phospholipid content, the invasomal dispersions’ higher viscosities would cause greater mass transfer resistances, lower sonication efficiency, and larger particles [32]. This result agrees with those previously published by Teaima et al. [31].

After analyzing the data, numerical optimization using Design-Expert software estimated the optimized formulation composition to maximize EE% and minimize vesicle size. The optimization design was found to be suitable and valid since the observed EE% and vesicle size of the optimized formulation were in good agreement with the predicted values. DSC is used to evaluate the crystallinity of CSN and to record any potential interactions between the CSN and other constituents of invasomes [32]. The disappearance of the characteristic peak of CSN in invasomes indicated that the drug is molecularly dispersed or present in an amorphous state and entrapped in the lipid bilayer of the invasomes. FT-IR spectra confirmed this conclusion, revealing that the optimized CSN-loaded invasomes exhibited the same spectra as their individual components, proving the successful incorporation of CSN within the vesicles and the absence of chemical interaction between the CSN, cholesterol, and phospholipid. It is more crucial to focus on the PDI of invasomes to evaluate the distribution of invasomal vesicles [40]. The PDI of CSN-loaded invasomes exhibited a monodisperse, homogeneous distribution of invasomes in the formulation. Zeta potential is a crucial indicator of the physical stability of the generated dispersions since it evaluates the degree of repulsion between adjacent vesicles [27, 32]. The zeta potential result revealed a negative value for the optimized formulation due to the presence of ethanol, which aids in the stabilization of the vesicular charges by electrostatic repulsion and reduces their eventual de-aggregation [27, 32, 57]. Electrostatic stabilization and non-aggregation of invasomal vesicles were confirmed by the TEM images. These results agree with those previously published by Tawfik et al. [32]. The sink condition was determined by obtaining an equilibrium solubility analysis to determine the saturation solubility of CSN and the volume of dissolution medium needed. To guarantee the sink condition of the release and permeation investigations, 50 mL of phosphate-buffered saline (pH 6.8) containing 0.1% (w/w) Tween 80 was chosen as the dissolution medium since it is larger than the saturation solubility of CSN. This result agrees with those previously published by Zerrin et al. [58]. It was shown through an in vitro drug release profile that the release rate of CSN from CSN-loaded invasomes was significantly higher than that of free CSN, demonstrating the solubilizing effect of invasomes leading to enhanced drug release and the aqueous solubility of CSN, and consequently improving its oral bioavailability. These findings verified that the crystalline structure of CSN was transformed into a soluble amorphous form during invasomal encapsulation, triggering the release of CSN. This result agrees with those previously published by Zerrin et al. [58]. The in vivo performance of nasal medication delivery systems is significantly associated with ex vivo drug permeation investigations [27, 32]. The synergistic effect of ethanol/cineole on CSN permeation may account for the increased penetration of CSN from optimized CSN-loaded invasomes [27, 32, 57]. The lipid bilayer becomes less stiff and more pliable due to the presence of cineole and ethanol, which improve its pliability, diffusion, and fluidity [29, 30]. This result agrees with those previously published by Salem et al. [27]. For simulating dissolution data, the Excel add-in DDSolver offers an alternative dissolution model for categorizing drug release kinetics using the model selection criterion (MSC), Akaike information criterion (AIC), and determination coefficient (*R*^2^) [27, 28]. By comparing these three factors, the Korsmeyer-Peppas model was found to be the most appropriate kinetic model for describing the release of CSN from a CSN-loaded invasomes. The “*n*” denotes the release exponent, which in turn represents the drug-release mechanism used in the formulation [59]. If *n* < 0.45, the drug is released via Fickian diffusion; *n* >  = 0.45, drug release is due to non-Fickian diffusion. The results of “*n*” provide evidence that CSN was released from a CSN-loaded invasomes by a mechanism of non-Fickian diffusion. Previous investigations on invasomes used different storage temperatures to assess their stability by measuring EE%, particle size, and PDI [29, 35, 60, 61]. At room temperature, invasomes demonstrate 50% more loss of encapsulated drug, as well as an increase in particle size and PDI value, indicating physical instability. There was no significant change in EE%, particle size, or PDI value in the invasomes after 3 months of refrigerator storage. Therefore, we advise keeping the optimized CSN-loaded invasomes formulation at 4 °C.

Diabetes mellitus stands out as a key contributor to atherosclerosis due to the hyperglycemia that accompanies it and leads to endothelial dysfunction, dyslipidemia, and insulin resistance [3, 5–8]. Five percent refined sugar was added to induce diabetes in rats and consequently hasten the induction of atherosclerosis [44]. Measurement of glucose level was used to confirm the successful induction of diabetes [45]. Increased serum glucose in the control positive rats in comparison to the control negative rats showed that we had successfully produced the experimental diabetes model in rats. A high-fat diet with vitamin D3 was added to induce hyperlipidemia, which leads to inflammatory cell infiltration, phagocytosis, and calcification of plaques, and consequently hastens the induction of atherosclerosis [44, 62, 63]. Decreased serum HDL and increased serum C, TG, LDL, and LDL and VLDL in the control positive rats in comparison to the control negative rats showed that we had successfully produced the experimental atherosclerosis model in rats. Success in inducing atherosclerosis in rats was confirmed by the presence of atherosclerotic plaques, foam cells, and calcium deposition as shown in histopathology. The results of the lipid profile reflected the anti-diabetic and anti-atherosclerotic activities of both free CSN and CSN-loaded invasomes. Additionally, atherosclerotic lesions were improved in the free CSN and CSN-loaded invasomes as shown in histopathology, when compared to the positive control group. These are because CSN is an effective vasodilator that increases blood flow on the arterial wall by decreasing NF-κβ, oxidative stress, plaque disruption, and macrophage formation in the aortas [14, 16, 17]. In addition, CSN inhibits insulin resistance and glucose intolerance, leading to a decrease in hyperglycemia [19]. Among treatment groups, the CSN-loaded invasome group had higher activity than oral CSN due to the solubilizing effect of invasomes leading to enhanced CSN release and the aqueous solubility of CSN, consequently improving its oral bioavailability. The synergistic effect of ethanol/cineole on CSN permeation may account for the increased penetration of CSN from optimized CSN-loaded invasomes [27, 32, 57]. Additionally, the surface area and network of blood vessels of the nasal cavity allow the drug to enter the systemic circulation directly without being subjected to first-pass metabolism [22–24]. Histopathology confirmed these results, where the CSN-loaded invasome group had the best improvement effect with a relatively intact aorta intimal structure. These results showed that treatment with nasal CSN-loaded invasome formulation has the potential to prevent the initiation and progression of atherosclerosis, particularly in diabetic patients. For this purpose, further clinical trials are recommended to confirm our promising point that we experienced in the diabetic atherosclerotic rats. Animals were subjected to toxicity tests so that we could learn more about the risks associated with nasal administering CSN-loaded invasomes and pinpoint any obvious side effects. A change in body weight is a good indicator of an animal’s overall health [48, 49]. During in vivo behavioral observations, all rats given CSN-loaded invasomes appeared healthy, with no deaths and no significant differences in body weight compared to the control negative group. This suggests that nasally administration of CSN-loaded invasomes had no discernible effect on normal growth and showed that no organs were adversely affected, nor did it show any signs of toxicity. Blood-related functions of CSN-loaded invasomes are a significant indication of physiological and pathological status that can be determined by hematological markers [48, 49]. The lack of a statistically significant variation in the RBC indices and WBC revealed that erythropoiesis, morphology, and osmotic fragility of red blood cells and the immune system were unaffected by the nasal delivery of CSN-loaded invasomes. Additionally, serum biochemistry was analyzed to see if the nasal delivery of CSN-loaded invasomes caused any changes in renal or hepatic function [48, 49, 64]. The lack of a statistically significant variation in the levels of ALT, AST, creatinine, and uric acid revealed that liver and kidney functions were unaffected by the nasal delivery of CSN-loaded invasomes. In conclusion, the results of this investigation showed that treatment with nasal CSN-loaded invasomes prevented the initiation and progression of diabetes-associated atherosclerosis in experimental rats.

## Conclusion

The current study aims to develop nasal CSN-loaded invasome drops to improve CSN’s permeation, release, and bioavailability as a potential treatment of diabetes-associated atherosclerosis. It was shown through an in vitro drug release profile that the release rate of CSN from CSN-loaded invasomes was significantly enhanced by 1.43, demonstrating the solubilizing effect of invasomes leading to enhanced drug release and the aqueous solubility of CSN, consequently improving its oral bioavailability. The ex vivo drug permeation investigations showed an increased penetration of CSN from optimized CSN-loaded invasomes by 1.94 due to the synergistic effect of ethanol/cineole. The in vivo results showed that nasal administration of CSN-loaded invasomes significantly increased serum HDL level and decreased serum glucose, C, TG, LDL, and VLDL levels, demonstrating its anti-diabetic and anti-atherosclerotic activities. In conclusion, the results of this investigation showed that treatment with nasal CSN-loaded invasomes prevented the initiation and progression of diabetes-associated atherosclerosis in experimental rats.

## Data Availability

Datasets used during this investigation can be obtained from the corresponding author.
